# 

**Published:** 1884-03

**Authors:** 


### CONTRIBUTORS.

| | |
|---|---|
| J. E. Blackshear, M. D | Macon, Ga. |
| W. D. Bizzell, M. D | Atlanta, Ga. |
| F. M. Brantley, M. D | Senoia, Ga. |
| A. W. Calhoun, M. D | Atlanta, Ga. |
| R. O. Cotter, M. D | Atlanta, Ga. |
| Hunter P. Cooper, M. D | New York. |
| J. D. S. Davis, M. D | Birmingham, Ala. |
| S. H. Dillard, M. D | Bellton, Ga. |
| A. S. Dyar, M. D | Atlanta, Ga. |
| W. S. Eklin, M. D | Atlanta, Ga. |
| W. H. Felton, M. D | Cartersville, Ga. |
| E. G. Ferguson, M. D | Macon, Ga. |
| Henry Gaither, M.D | Oxford, Ga. |
| J. McF. Gaston, M. D | Atlanta, Ga. |
| N. G. Gewinner, M. D | Macon, Ga. |
| W. C. Gibson, M. D | Macon, Ga. |
| James A. Gray, M. D | Atlanta, Ga. |
| S. H. Gray, M. D | Barnesville, Ga. |
| W. W. Greer, M. D | Atlanta, Ga. |
| A. W. Griggs, M. D | West Point, Ga. |
| C. H. Hall, M. D | Macon, Ga. |
| W. A. Hammond, M. D | New York. |
| L. G. Hardman, M. D | Harmony Grove, Ga. |
| Sidney Herbert | Atlanta, Ga. |
| C. W. Hickman, M. D | Augusta, Ga. |
| W. F. Holt, M. D | Macon, Ga. |
| J. G. Hopkins, M. D | Thomasville, Ga. |
| D. H. Howell, M. D | Atlanta, Ga. |
| J. M. Hull, M. D | Augusta, Ga. |
| Seth N. Jordan, M. D | Columbus, Ga. |
| W. A. Love, M. D | Atlanta, Ga. |
| H. McHatton, M. D | Macon, Ga. |
| T. M. McIntosh, M.D | Thomasville, Ga. |
| H. V. M. Miller, M. D., LL. D | Atlanta, Ga. |
| K. P. Moore, M. D | Macon, Ga. |
| F. H. Nichols, M. D | Atlanta, Ga. |
| G. H. Noble, M. D | Atlanta, Ga. |
| W. B. Parks, M. D | Atlanta, Ga. |
| C. C. Quillian, M. D | Atlanta, Ga. |
| Thomas L. Raines, M. D | Atlanta, Ga. |
| C. A. Lee Reed, M. D | Cincinnati. |
| E. F. Starr, M. D | Narcoochee, Ga. |
| J. P. Stevens, M. D | Macon, Ga. |
| J. S. Todd, M. D | Atlanta, Ga. |
| Geo. R. West, M. D | Rome, Ga. |
| W. F. Westmoreland, M. D | Atlanta, Ga. |
| H. J. Williams, M. D | Macon, Ga. |
| J. D. Wilson, M. D | Atlanta, Ga. |
| L. B. V. Wooley, M. D | Atlanta, Ga. |
| T. J. Word, M. D | Atlanta, Ga. |

### OUR ADVERTISERS.

We desire to call special attention to the advertisements in this number of the JOURNAL; they are from the best and most reliable houses that deal in medicines, instruments and surgical appliances.

*Magnus & Hightower.*—We know these gentlemen personally, and can confidently endorse them as being strictly reliable in their dealings. Physicians and others entrusting their orders to them may rest assured that they will be honestly and squarely dealt with. See their advertisement.

*Hernstein.*—This old established instrument maker has an advertisement in this number of the JOURNAL. Recognizing the wants of the Southern physicians, he has established a branch house in this city, where any instrument or appliance can be had. If what is wanted is not in stock, Mr. Phillips, the gentlemanly manager, will have it made in the shortest time possible.

*Rio Chemical Company.*—This company manufacture extensively Celerina and Pinus Canedensis, two remedies that have an extensive sale. See their advertisement.

*Battle & Co.*—This enterprising St. Louis firm manufacture a number of new compounds. Their remedies are well sustained by chemical experience; especially is this true of Bromidia and Iodia.

*F. De Bary & Co.*—These gentlemen are sole agents for the United States for the justly celebrated Apollinaris and the Hunyadi Janos mineral waters.

*Reed & Carnrick.*—This reliable New York house was the first to introduce to the profession the justly celebrated Beef Peptonoids. It is a concentrated powdered extract of beef partially digested, and combined with an equal portion of gluten.

*Maltine.*—This concentrated extract of malted barley, wheat and oats maintains its high reputation, notwithstanding its many rivals. It may be procured plain, or combined with tonics and alteratives, and we have found most beneficial results from some of the latter combinations. The extract seems to assist the alterative action of a number of medicines.

*E. Fougera & Co.* make a specialty of importing the best class of foreign medical preparations, and have been extraordinarily successful in building up a trade in such goods. Everything they advertise is the best of its class.

*Bellevue Hospital Medical College.*—No medical college stands higher with Southern physicians than does this one. Attention is directed to its advertisement.

*Bromo Chemical Co.*—This company manufacture quite a number of new medicines, one of which is Bromo Chloralum, a remedy that they claim a great deal for in scarlet fever. See their advertisement.

*Anglo-Swiss Milk Food.*—The fact that this food is used in the New York Infant Asylum by Dr. J. Lewis Smith should recommend it.

THE NEW YORK POLYCLINIC, A SCHOOL OF CLINICAL MEDICINE AND SURGERY FOR PRACTITIONERS.—This school has taken a prominent position among medical institutions in New York, as well as in the United States and Canada. It is, without doubt, the strongest clinical organization on this continent. Its faculty hold seventy official professional positions in the hospitals, asylums and dispensaries of the metropolis, and this vast material is utilized for the benefit of practitioners who desire to study special clinical medicine and surgery. The success of the Polyclinic has been remarkable. One hundred and thirty physicians have studied in the various classes since October, 1883, making a total of 291 within the first fifteen months of the existence of this institution. The class is divided into sections, so that the demonstration of cases is in the immediate presence of the members of the section. *None but practitioners are admitted.* The managers have recently purchased a magnificent property, which is now being thoroughly fitted up as a school and hospital. We call the especial attention of physicians wishing to visit the East for purposes of "finishing up," or for special study, to the advertisement of the Polyclinic on another page.

### NOTICE.

Original communications solicited. When articles require illustration the engravings will be furnished by the publishers free of charge.

SUBSCRIPTION: $2.50 a year.

Subscribers will please not send us money by Postal Notes. Remit by Express, Draft, Post-office Order or Registered Letter.

Address all letters and books and make all drafts payable to

THE ATLANTA MEDICAL AND SURGICAL JOURNAL,
Care Jas. P. Harrison & Co., Atlanta, Ga.

